# Organizational contextual features that influence the implementation of evidence-based practices across healthcare settings: a systematic integrative review

**DOI:** 10.1186/s13643-018-0734-5

**Published:** 2018-05-05

**Authors:** Shelly-Anne Li, Lianne Jeffs, Melanie Barwick, Bonnie Stevens

**Affiliations:** 1 Lawrence S. Bloomberg Faculty of Nursing, University of Toronto, Toronto, ON Canada; 2 St Michael’s Hospital Volunteer Association Chair in Nursing Research, Li Ka Shing Knowledge Institute, Toronto, ON Canada; 3 Lawrence S. Bloomberg Faculty of Nursing and Institute of Health, Policy Management and Evaluation, University of Toronto, Toronto, Canada; 4 Child Health Evaluative Sciences, Research Institute, Peter Gilgan Centre for Research and Learning, The Hospital for Sick Children, Toronto, ON Canada; 5 Department of Psychiatry, University of Toronto, Toronto, Canada; 6 The Dalla Lana School of Public Health, University of Toronto, Toronto, Canada; 7 Faculties of Medicine and Dentistry, University of Toronto Centre for the Study of Pain, University of Toronto, Toronto, Canada

**Keywords:** Organizational context, Implementation, Knowledge translation, Evidence-based practice, Healthcare, Adoption, Organization, Context, Integrative review

## Abstract

**Background:**

Organizational contextual features have been recognized as important determinants for implementing evidence-based practices across healthcare settings for over a decade. However, implementation scientists have not reached consensus on which features are most important for implementing evidence-based practices. The aims of this review were to identify the most commonly reported organizational contextual features that influence the implementation of evidence-based practices across healthcare settings, and to describe how these features affect implementation.

**Methods:**

An integrative review was undertaken following literature searches in CINAHL, MEDLINE, PsycINFO, EMBASE, Web of Science, and Cochrane databases from January 2005 to June 2017. English language, peer-reviewed empirical studies exploring organizational context in at least one implementation initiative within a healthcare setting were included. Quality appraisal of the included studies was performed using the Mixed Methods Appraisal Tool. Inductive content analysis informed data extraction and reduction.

**Results:**

The search generated 5152 citations. After removing duplicates and applying eligibility criteria, 36 journal articles were included. The majority (*n* = 20) of the study designs were qualitative, 11 were quantitative, and 5 used a mixed methods approach. Six main organizational contextual features (organizational culture; leadership; networks and communication; resources; evaluation, monitoring and feedback; and champions) were most commonly reported to influence implementation outcomes in the selected studies across a wide range of healthcare settings.

**Conclusions:**

We identified six organizational contextual features that appear to be interrelated and work synergistically to influence the implementation of evidence-based practices within an organization. Organizational contextual features did not influence implementation efforts independently from other features. Rather, features were interrelated and often influenced each other in complex, dynamic ways to effect change. These features corresponded to the constructs in the Consolidated Framework for Implementation Research (CFIR), which supports the use of CFIR as a guiding framework for studies that explore the relationship between organizational context and implementation. Organizational culture was most commonly reported to affect implementation. Leadership exerted influence on the five other features, indicating it may be a moderator or mediator that enhances or impedes the implementation of evidence-based practices. Future research should focus on how organizational features interact to influence implementation effectiveness.

**Electronic supplementary material:**

The online version of this article (10.1186/s13643-018-0734-5) contains supplementary material, which is available to authorized users.

## Background

Each year, at least $160 billion is allocated to medical and health research expenditures in North America [1, 2]. Despite major financial investments and advancements in knowledge generation for evidence-based practices (EBPs), healthcare organizations encounter significant implementation failures or challenges [3]. EBP entails making decisions about how to provide or promote healthcare by integrating the best available research evidence with clinical expertise and patient values and preferences [4]. A variety of definitions for the term “implementation” exists in health research. In this review, implementation is defined as “the use of strategies to adopt and integrate evidence-based health interventions and change practice patterns within specific settings” [5]. The estimated average evidence-to-practice time lag is 17 years [6]. This “know-do” gap can result in suboptimal care or a delay in benefits associated with unsuccessful implementations [7]. While provider-level characteristics such as knowledge, attitudes, and behavior about the EBP are widely acknowledged to be critical in addressing this know-do gap, organizational contextual features have also been recognized as a key consideration when implementing EBPs in healthcare settings [7–9]. Over the last decade, addressing this gap has been a priority research focus in implementation science. One such focus has been the need to better understand the role organizational contextual features play in supporting or hindering implementation [10, 11].

Currently, there are multiple definitions for the term “organizational context” in various disciplines. Quality improvement (QI) literature appears to establish parameters around this term. Glasgow et al. [12] developed an analytic framework to describe how organizational context modifies QI. The authors described how the intrinsic organizational features such as staffing and culture, facility structure, and QI experience together make up the organizational context of a QI initiative. Extant organizational management literature appears to have the most mature conceptualization of organizational context, often including components such as organizational culture, climate, goals and missions, processes (policies, mode of governance), power dynamics, state/condition, structure (size, shape and type of organization, hierarchical levels), and time [12–14]. Context is commonly depicted in three levels, and researchers tend to reserve the term “organizational context” for internal organizational features. The macro level recognizes the influence of political-economic forces, which focuses on interactions between markets and societies at the broadest level. The meso level represents organizational characteristics such as culture, climate, tacit rules, and shared meanings that influence individual behaviors [15, 16]. The micro level consists of activities in the local setting that provide a contextual influence. Together, these levels of context form a complex set of influences on organizations [15].

The relationship between implementation outcomes and context have been described in implementation theories, models, and frameworks including Rogers’ diffusion of innovations theory [17]; the Consolidated Framework for Implementation Research framework (CFIR) [18]; the Exploration, Preparation, Implementation, and Sustainment framework [19]; the Integrated Promoting Action Research in Health Services framework (i-PARiHS) [20]; and the Theoretical Domains Framework [21]. The implementation theories, models, and frameworks appear to characterize context as a multi-dimensional concept that interacts with different phases of knowledge translation (KT).

### Problem identification

While these implementation frameworks include context, no single framework is sufficiently comprehensive about what comprises context. In addition, the authors of the frameworks are often inconsistent in how context is theoretically and operationally defined. Without a shared understanding of context and its characteristics and features, there is little direction to which features of context are most influential to KT efforts [22]. Extending beyond conceptual theories, models, and frameworks; this review aims to synthesize and summarize organizational contextual features commonly reported to influence the implementation of EBPs in actual healthcare settings.

## Methods

The guiding question for the review was the following: Which organizational contextual features are most commonly reported to influence the implementation of EBP in healthcare settings? Studies with diverse study designs and methods (qualitative, quantitative, mixed methods) that explored, described, or measured organizational contextual features in implementation research were included in this review [23]. Only empirical literature was included. Methodological rigor was informed by Whittemore and Knafl’s [24] five-phase integrative review method: problem identification (noted above), literature search, data evaluation, data analysis, and result presentation.

### Literature search

The search strategy (see Additional file 1) was developed on MEDLINE in consultation with two librarians and applied to Cochrane databases, CINAHL, MEDLINE, EMBASE, PsycINFO, and gray literature. The journal *Implementation Science* (from journal inception to June 2017) was hand searched to uncover additional relevant articles. The search included four categories of search key terms: (a) Implementation, (b) Context, (c) Evidence, and (d) Organization. Inclusion and exclusion criteria (Table 1) were applied during screening.

**Table 1:** Inclusion and exclusion criteria

Inclusion criteria	Exclusion criteria
Articles were included if they:	Articles were excluded if they:
Published in a peer-reviewed journal	Were outside the healthcare domain
Investigated contextual features at the organizational level as a primary or secondary study objective	Did not investigate a KT initiative Were editorials, opinions, conceptual papers, discussions, or textbooks
Focused on implementation (including adoption, uptake, and research utilization)	Were reported in languages other than English
Empirical studies of all design types; Were published since 2005, to capture a wave of research on organizational context over the past 12 years [78–80]	Did not report on any organizational contextual feature in the findings and discussion sections of the published report

### Data evaluation

Following the removal of duplicates, two reviewers (SAL, PEA) independently double-screened the titles and abstracts of a randomly selected sample (generated from an online randomized website) of 20% (*n* = 1034) of the retrieved citations to ensure interrater reliability. Once suitable agreement (*k* = 0.85) was achieved, title and abstract screening was undertaken for all citations. Citations missing an abstract during screening were retained for full-text screening to establish eligibility. Citations meeting eligibility criteria were included for full-text screening. Full-text screening followed the same strategy as the title and abstract screening to ensure interrater reliability.

Data extraction was performed by the same reviewers (SAL, PEA) for all included articles, independently and in duplicate. A third reviewer was available to resolve any disagreement between the two reviewers; however, all disagreements were resolved via consensus without involving a third reviewer.

### Quality appraisal

The Mixed Methods Appraisal Tool (MMAT [25]) guided the quality appraisal for all qualitative, quantitative, and mixed methods studies. Quantitative and qualitative studies were each assessed by four criteria with overall scores varying from 0% (no criterion met) to 100% (all four criteria met). For mixed methods studies, three components were appraised: qualitative, quantitative, and mixed methods component, with the overall score determined by the lowest component score. In keeping with integrative review methods [24, 26], all records were retained in the analysis, regardless of score. For each article, two reviewers assessed methodological quality independently and discrepancies were resolved via consensus.

### Data analysis

For each study, the steps of data reduction, data display, data comparison, and drawing conclusions and verifications were followed [24]. To ensure trustworthiness and rigor during data abstraction and synthesis, a table was developed to summarize the organizational contextual features. The abstracted information was compared, and patterns of findings were recorded as they emerged, followed by groupings of similar data and the identification of several key themes [24].

Analysis followed a qualitative descriptive approach, given that most of the study designs were qualitative and the results from quantitative studies could not be aggregated due to heterogeneity in study design, outcomes, and type of intervention [27]. Inductive content analysis was used to uncover themes related to organizational context [28, 29]. This analytic approach involved reading and rereading the articles to uncover any salient codes and categories, subsequently collapsing them into themes (organizational contextual features) [27].

## Results

**Fig. 1:**
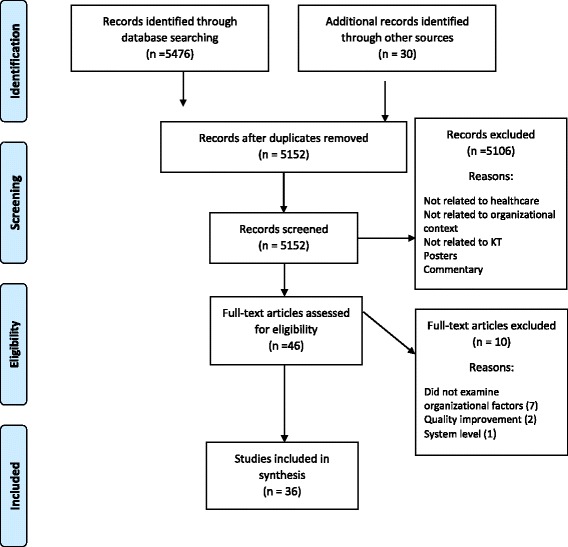
Flow diagram of selected studies

Figure 1 depicts the search and screening phases as per Preferred Reporting Items for Systematic Reviews and Meta-Analyses (PRISMA) guidelines [30]. The search yielded 5152 citations. Following a review of titles and abstracts, 46 relevant articles were retrieved in full text and reviewed for eligibility. Of these, 10 were excluded because they did not explicitly explore and/or measure organizational context (*n* = 7), were part of quality improvement but did not include implementation of EBPs (*n* = 2), or were based on a system-level study (*n* = 1). Thirty-six peer-reviewed journal articles were included in the integrative review. The studies were methodologically diverse; 11 (30.6%) were quantitative studies that explored organizational context as an outcome using cross-sectional surveys, 20 (55.6%) were qualitative studies that described organizational context using themes derived from interviews and/or focus groups, and 5 (13.9%) were mixed methods studies. Even though implementation success was frequently mentioned in the included studies, none defined implementation success.

### Description of studies

Table 2 presents the characteristics of the included studies, including study setting, study aim, sample, guiding framework (if applicable), study design and data collection methods, main findings, and MMAT quality score. The studies were published between 2007 and 2017 and were based in 11 countries. At least 8094 participants were included in this review. Study participants comprised a wide range of stakeholders including physicians, nurses, and allied health professionals working as coordinators, medical staff, and senior managers from many different healthcare settings. Two reported on the number of participating pediatric hospital units (*n* = 16) and medical centers (*n* = 12) instead of the number of individual participants. Twenty (56%) of 36 studies used a theory, model, or framework to guide their data collection and/or analysis.

**Table 2:** Characteristics of included studies

Authors, date, country, setting	Study aim	Sample (*N*)	Guiding framework	Design, data collection method	Main findings	MMAT Quality score (0–100)
Abdekoda et al. (2015) Iran University-affiliated hospitals	To determine organizational contextual factors that may affect physicians’ acceptance of electronic medical record’s (EMR) adoption	Physicians (general practitioners, specialists, clinical fellows) (330)	Technology acceptance model	Quantitative; cross-sectional surveys	Organizational contextual factors are main determinants in leading physicians’ attitude toward EMRs adoption	100
Barnett et al. (2011) UK Primary and secondary healthcare organizations	To explore how healthcare innovators of process-based initiatives perceived factors that promoted or hindered innovation implementation and diffusion	Representatives of organizations who were winners of innovation in healthcare award (15)	None	Qualitative; semi-structured interviews	Inter-organizational partnerships human resources (champions) were integral in developing, establishing and diffusing the innovations	75
Bergstrom et al. (2012) Uganda Health centers that provide obstetric services	To examine relevance of organizational context from PARiHS, and whether other factors organizational context was perceived to influence implementation strategies for low-incoming settings from the perspectives of midwives and managers	Nurses, midwives, physicians (23)	PARiHS	Qualitative; semi-structured interviews and focus groups	Receptive context, culture, leadership, access to resources, community and evaluation—are relevant to influencing implementation efforts	75
Berta et al. (2010) Canada Long-term care settings	To enhance understanding of what enables or impedes a health care organization when applying new knowledge intended to improve care in long-term care (LTC)	Administrative staff, clinical staff (63)	Organizational learning theory	Qualitative; semi-structured interviews and focus groups	Organizational contextual elements essential for successful knowledge application. Leaders vital in the success of knowledge application processes	75
Carljford et al. (2010) Sweden Primary healthcare units	To identify key factors influencing the adoption of an innovation being introduced in primary healthcare units in Sweden	General practitioners, nurses, nursing assistants, dietitians, welfare officers, occupational therapists (67)	Rogers’ diffusion of innovations	Qualitative; focus groups	Adoption positively influenced by perceptions of the innovation being compatible with existing routines and norms. Organizational changes and staff shortages can be obstacles for adoption process	75
Chuang et al. (2011) USA Various healthcare organizations	To better understand the organizational and relational factors that influence middle managers’ support for the innovation implementation process	Middle managers across various healthcare organizations (92)	Organizational framework of innovation implementation	Qualitative; semi-structured interviews and focus groups	There is interplay between middle managers’ control and discretion, and the dedication of staff and other resources for empowering managers to implement the complex innovation	75
Cummings et al. (2010) Canada Hospitals	To elicit pediatric and neonatal healthcare professionals’ perceptions of the organizational context in which they work and their use of research to inform practice	Registered nurses (RN), nurse practitioners (NP), graduate nurses (GN) (248)	PARiHS	Quantitative; cross-sectional surveys	Nurses in contexts with more positive culture, leadership, and evaluation reported more research utilization than nurses in less positive contexts	100
Doran et al. (2012) Canada Hospitals, long-term care (LTC) facilities, and community organizations	To investigate the role of organizational context and nurse characteristics in explaining variation in nurses’ use of personal digital assistants (PDAs) and mobile Tablet PCs for accessing evidence-based information	RN, NP in long-term care (469)	PARiHS	Quantitative; cross-sectional surveys	Frequency of best practice guideline use was explained by resources, organizational time, staffing. Frequency of Nursing Plus database use explained by culture, resources, breadth of device functions	100
Estabrooks et al. (2007) Canada and USA US army hospitals, Canadian hospital healthcare settings	To compare research utilization in two different healthcare contexts—Canadian civilian and US Army settings.	RN, NP, nurse managers (1750)	None	Mixed methods; self-report surveys, interviews, observational study	Predictors in the US Army setting for research use: trust and years of experience; and Canadian civilian setting: in-service attendance, time (organizational), champion, library access	75
Estabrooks et al. (2008) Canada Acute care hospitals	To examine the determinants of research use among nurses working in acute care hospitals, with an emphasis on identifying contextual determinants of research use	RN, NP (235)	Rogers’ diffusion of innovations	Quantitative; cross-sectional surveys	Units with highest mean research utilization scores clustered on unit culture, importance of continuing education, environmental complexity. Lowest research use scores clustered on high workload and lack of people support	75
Estabrooks et al. (2015) Canada Nursing homes	To investigate the influence of individual and organization context factors on use of best practices by care aides in nursing homes in the Canadian prairie provinces	Nursing home facilitators, home care aides, managers (1282)	None	Quantitative; cross-sectional surveys	Significant predictors were evaluation (feedback mechanisms), structural resources, and organizational slack (time) for best practice use by care aides	100
Green et al. (2017) England Acute medical units	To investigate the implementation of two distinct care bundles in the acute medical setting and identify the factors that supported successful implementation		CFIR	Qualitative; review of recorded meeting minutes and audio recordings of meetings	Resources to support initiatives (incl. training), perceived sustainability of changes, senior leadership support was seen as critical	75
Harris et al. (2013) USA Outpatient medical clinics	To explore the organization contextual factors that were important for implementation of a short message system (SMS)-based intervention for persons living with Human Immunodeficiency Virus (HIV)	Providers, study coordinator, patients (14)	Weiner et al.’s [81] conceptual model of process evaluation	Qualitative; in-depth interviews	Leadership and resources important in implementing SMS based intervention	75
Harvey et al. (2015) UK Health service organizations	To extend and develop an understanding of how organizational context affects the implementation and effectiveness of improvement in healthcare organizations	Middle-level and senior-level managers in hospitals (22)	Absorptive Capacity Framework	Qualitative; semi-structured interviews	Strategic priorities, communication resources on learning, collaboration with external stakeholders and make use of available knowledge important for implementation success.	50
Hofstede et al. (2013) Netherlands General hospitals, medical centers, private clinics	To explore and categorize all barriers and facilitators associated with the implementation of shared decision making in sciatica care from the perspectives of healthcare providers and patients	Physical therapists, surgeons, general practitioners, neurologists (62)	Grol and Wensing’s [82] model	Qualitative; semi-structured interviews and focus groups	Lack of time, high workload, lack of trust, and communication issues were barriers to implementation	50
Koehn et al. (2008) USA Large, urban medical center	To investigate registered nurses’ perceptions, attitudes and knowledge/skills associated with evidence-based practice	RN, NP (422)	None	Quantitative; cross-sectional surveys	Lack of time, leadership buy-in, and resources as main barriers. Implementing culture of EBP important to moderate staff attitudes on EBP uptake	75
Krein et al. (2010) USA Hospitals	To examine quality improvement efforts and the implementation of recommended practices to prevent central line-associated bloodstream infections (CLABSI) in US hospitals	Epidemiologists, nurses, physician directors, front-line clinicians (86)	Rogers’ diffusion of innovations	Qualitative; semi-structured interviews	Type of cultural, emotional and political context greatly affect implementation. Collaboration, leadership and resources play key role in uptake	75
Livet et al. (2008) USA Mental health centers	To examine the organizational-level mechanisms that are part of the Prevention Delivery System and their influence on implementation of comprehensive programming frameworks aimed to help practitioners plan, implement, evaluate and sustain their interventions	Board and provider agency representatives (32)	None	Quantitative; cross-sectional surveys and interviews (coded and quantified)	Leadership, shared vision, champions, technical assistance (resources) were common correlates of use across programming processes	100
Lodge et al. (2016) USA State hospitals, community centers	To identify barriers to implementing a person-centered recovery planning system for mental health patients.	Leadership, case managers, rehabilitation specialists, social workers, psychologists, coordinators (71)	CFIR	Qualitative; focus groups	Lack of time and resources (incl. training), lack of staff buy-in, non-collaborative planning, leadership barriers, dissemination barriers related to implementation failure	50
Marchionni et al. (2008) Canada Inpatient units in a large healthcare center	To examine what contextual factors support the implementation of best practice guidelines (BPG) in nursing care	RN, NP (20)	None	Quantitative; pre and post design surveys	Supportive organizational culture and key people leading change important for implementing BPG	75
McCullough et al. (2015) USA Anticoagulation clinics	To identify the interconnected patterns among contextual elements that influence uptake of an anticoagulation clinic improvement initiative	Pharmacy administrators, pharmacists, nurses, support staff (51)	PARiHS	Qualitative; semi-structured interviews, ethnographic observations	Leadership, teamwork and communication interacted with each other, often yielding results that could not be predicted by looking at just one factor alone	75
Olstad et al. (2011) Canada Recreational facilities	To investigate the awareness, adoption and implementation of a nutritional guideline for children among recreational facilities	Mayors, councilors, middle-level managers (151)	Greenhalgh’s multi-tiered model of diffusion of complex innovations, Prochaska and Velicer’s transtheoretical model of change	Mixed methods; cross-sectional survey with open- and close-ended questions	Inner context, negative feedback received during the implementation process, managers’ belief that implementing nutrition guidelines would limit profit were key barriers to uptake	50
Omer et al. (2012) Saudi Arabia Large hospitals	To explore barriers to and facilitators of research finding utilization in nursing practice	Nurses, nursing managers (413)	None	Quantitative; cross-sectional surveys	Communication, adopter, and innovation factors; lack of time, lack of authority, lack of physician cooperation, lack of EBP-related education are barriers to research use	100
Ozdemir and Akdemir (2009) Turkey Inpatient clinics in hospitals	To identify the factors that the nurses believe are essential for evidence to become the basis of their practice and the obstacles to research utilization	RN, NP (219)	None	Quantitative; cross-sectional surveys	Older and highly experienced nurses likely to implement evidence into practice; research use related to organizational support	75
Powell et al. (2009) UK Acute care hospitals	To explore organizational difficulties during the implementation of national policy recommendations in local contexts.	Anesthetists, surgeons, nurses, managers (71)	None	Qualitative; case-study; semi-structured interviews	Networks, financial resources, time and training affected local uptake of national policy recommendations	75
Riekerk et al. (2009) Netherlands Intensive care unit in a teaching hospital	To implement a delirium screening instrument into daily critical care, to assess the obstacles to its implementation.	Physicians, nurses (53)	None	Quantitative; pre-post surveys	Communication, staffing and training emerged as important elements for implementation	50
Sommerbakk et al. (2016) Norway Local medical centers (primary care services that offer short-term in-patient care)	To determine the barriers and facilitators for implementing improvements in PC have been experienced by health care providers	Physicians, nurses, managers (20)	Grol and Wensing’s (2004) model	Qualitative; semi-structured interviews and focus groups	Barriers and facilitators were connected to: credibility, advantage, accessibility of innovation; individual motivation, PC expertise, confidence; patient compliance; leadership, culture, communication, resources, expertise, policy, finance, training, reminders	75
Squires et al. (2013) Canada Medical, surgical, critical care units in pediatric hospitals	To identify dimensions of organizational context and individual (nurse) characteristics that influence pediatric nurses’ self-reported use of research	RN, NP (735)	None	Mixed methods; semi-structured interviews, non-participant observation, document analysis, cross-sectional survey	Predictors of conceptual research use: belief suspension-implement, problem solving ability, use of research in the past, leadership, culture, evaluation, formal interactions, informal interactions, organizational slack-space, and unit specialty	100
Stevens et al. (2014) Canada Pediatric hospitals	To determine the effectiveness of the KT strategies implemented in relation to unit aims; describe KT strategies implemented and their influence on pain assessment and management practices across unit types; identify facilitators and barriers to the implementation of KT strategies	Pediatric hospital units (16)	None	Mixed methods; chart review; process evaluation checklist (analyzed with qualitative content analysis)	Unit leadership, staff engagement, dedicated time and resources facilitated effective implementation of KT strategies.	75
Thomas et al. (2011) UK National health service organizations	To identify organizational factors facilitating research-based practice in allied health profession departments.	Clinicians and operational managers (58)	None	Qualitative; semi-structured interviews	Staff development, communication, resources and infrastructure, evaluation and feedback facilitated research use	
Urquhart et al. (2014) Canada Women’s and children’s hospital	To examine the key interpersonal, organizational, and system level factors that influenced the implementation and use of synoptic reporting tools in three specific areas of cancer care	Radiologists, endoscopists, surgeons (53)	PARiHS, organizational framework of innovation implementation (Helfrich et al. [83])	Qualitative; semi-structured interviews, document analysis, non-participant observation	Stakeholder involvement, communication, training and support, champions and respected colleagues, administrative and managerial support, and innovation attributes influential to implementation initiative	75
Vamos et al. (2017) USA Hospitals	To explore the multilevel contextual factors that influenced the implementation of the Obstetric Hemorrhage Initiative (OHI) among hospitals	Multidisciplinary hospital staff (50)	CFIR	Qualitative; individual in-depth interviews	Leadership engagement; engaging people; planning; reflecting, inner staff knowledge/beliefs; resources; communication; culture. Leadership and staff buy-in emerged as important components influencing OHI implementation across disciplines	75
Whitley et al. (2009) USA Mental health centers	To examine which factors promote or hinder successful implementation of illness management and recovery in these settings	Mental health centers (12)	None	Mixed methods; semi-structured interviews, field notes, cross-sectional surveys	Leadership, culture, training, staff and supervision meaningfully determined implementation success/failure. These themes worked synergistically to effect implementation	75
Wright et al. (2007) UK Rehabilitation units	To identify the contextual indictors that enable or hinder effective evidence based continence care in rehabilitation settings for older people	Medical staff, nursing leaders, nursing staff (123)	PARiHS	Mixed methods; self-reported surveys, semi-structured observation of practice	Leadership, evaluation and culture barriers led to poor uptake	75
Yamada et al. (2017) Canada Pediatric hospitals	To assess how organizational context moderates the effect of research use and pain outcomes in hospitalized children.	RN, NP (779)	None	Quantitative; cross-sectional surveys	Culture, social capital, informal interactions, resources, organizational slack significantly moderated the effect of instrumental research use on pain assessment; culture, social capital, resources and organizational slack time moderated the effect of conceptual research use and pain assessment	100
Zazzali et al. (2008) USA Mental health service organizations	To explain the adoption and implementation of FFT in a small sample of family and child mental health services organizations	Administrators (15)	None	Qualitative; semi-structured interviews	Resource, organizational structure and culture influenced the ease with which treatment program was implemented	75

*Note*: MMAT scores vary from 25% (one criterion met) to 100% (all criteria met). For qualitative and quantitative studies, this score is the number of criteria met divided by four. For mixed methods studies, the overall quality score is the lowest score of the quantitative and qualitative study component

### Methodological quality

The included studies were of moderate to high methodological quality (Table 1) based on the MMAT [25] appraisal. Of the 36 studies, 22 received a score of 75% (moderately high quality), 8 received 50% (moderate quality), and 5 received 100% (high quality).

### Organizational contextual features in empirical studies

Six organizational contextual features included organizational culture; networks and communication; leadership; resources; evaluation, monitoring, and feedback; and champions. A series of sub-features included collaboration, teamwork, communication, financial resources, time, staffing and workload, and education and training. Table 3 illustrates the features and sub-features.

**Table 3:** Number of studies that reported on each feature, and their corresponding references

Features and sub-features	Number of studies out of 36	Reference
Organizational culture	22	[3, 31–36, 38–43, 49, 51–53, 56, 84, 85];
Networks and communication	22	[3, 31, 33, 36–38, 40, 42, 43, 45–50, 52–54, 58, 85, 86];
Leadership	20	[32–34, 37–40, 42–45, 48–50, 52, 55, 56, 84, 85];
Resources		
Financial resources	17	[3, 33, 35–37, 44, 46, 50, 52, 53, 54–56, 59, 84, 86];
Staffing and workload	14	[32, 35, 36, 38, 41, 43, 47–50, 57, 84];
Time	13	[35, 37, 42, 48, 51, 53, 55, 57–59, 85, 86];
Education and training	12	[32, 33, 37–39, 46, 48, 51–54, 56, 84];
Evaluation, monitoring, and feedback	14	[31, 32, 36, 39, 42–44, 48, 49, 51, 52, 56, 85];
Champion	11	[3, 32, 33, 36, 44, 48, 49, 52, 56, 59];

### Organizational culture

Organizational culture was included as an organizational contextual feature in 22 of 36 (61%) studies. Organizational openness to trialing new innovations and a learning culture were highly associated with implementation success [30–35]. Conversely, an absence of a learning culture can act as a major hindrance to successful implementation [36]. Organizational cultures comprised of staff who have too much autonomy (i.e., physicians experiencing a high level of autonomy when making decisions about how to treat patients) [37], are resistant to trial new innovations [38], or are unclear about organizational values and beliefs [39] can be barriers to successful implementation. Sites demonstrating high implementation fidelity were marked by a strong culture of innovation, accompanied by positive staff attitudes and behavior toward the new initiative [40]. In one study, unit culture (measured by work creativity, work efficacy, questioning behavior, co-worker support, and emphasis on continuing education) was a significant predictor of nurses’ research use [41]. These results corroborated with other studies investigating organizational context and nurses’ research utilization [32, 42]. Organizational culture significantly moderated the effect of nurses’ instrumental (direct use of research knowledge) and conceptual research use (indirect use of research knowledge) on pediatric pain assessment in hospitals [43].

### Networks and communication

An association of organizational networks and communication with implementation success was evident in 22 of 36 studies (61%). Three sub-features were commonly associated with implementation outcomes.

### Collaborations

Collaborative relationships that occur within and external to the organization were important for carrying out implementation plans. For instance, Barnett et al. highlighted two main purposes of interorganizational collaborations. First, materially based partnerships provided the organization with the resources required for the implementation and diffusion of new programs. Second, symbolically based interorganizational collaborations allowed organizations to gain local consensus to bolster the new programs with legitimacy, which in turn serves as an important social exchange that assisted with communicating the innovation’s impact through gaining a broader consensus. Harvey et al. [39] described how close collaboration with an external implementation improvement team can support staff and leadership development geared toward implementing change.

### Teamwork

Teamwork was characterized as good working relations, the ability to communicate clearly and effectively, and the capacity to solve problems together during EBP uptake [44–47]. Using an ethnographic case study design, McCullough et al. [47] observed that strong teamwork among staff, when combined with strong belief in evidence, led to high adoption of a dosing algorithm in anticoagulation clinics. However, when staff were dismissive of the evidence, strong teamwork served to reinforce resistance to implementation efforts. Teamwork was highly relevant in new programs that required participation from professionals in multidisciplinary teams. In a mental health organization where multidisciplinary staff (peer specialists, practitioners) were required to implement a new person-centered recovery program for their patients, poor multidisciplinary teamwork resulted in poor program uptake [38].

### Communication

Communication greatly impacted the implementation of EBPs [37, 39, 44, 46, 48–50]. The establishment of systems and processes to more effectively manage information and communication about the change initiative influenced implementation success [39]. Communication between healthcare professionals in a Dutch intensive care unit (ICU) was an important barrier for a successful start of the implementation phase of a delirium scoring system [48]. Vamos et al. [50] and Stevens et al. [49] articulated various communication channels that facilitated implementation in hospital units, including active (scheduled meetings, debriefings, emails) and passive (flyers, announcements on bulletin boards, auto-generated reminders) communications.

### Leadership

Leadership was reported in 20 of 36 (56%) studies as an important feature for implementation effectiveness. Leaders were often seen as providers of new knowledge and as key influencers in new implementation initiatives [44]. Leaders who created environments with high staff morale allowed staff to perceive themselves as part of the implementation team. Transformational leadership often gave rise to clear roles and effective teamwork structures and cultivated a culture of learning [44]. Leaders ensured changes were sustained, without which staff were reported to “fall back into the old ways of doing things” [37]. Senior leaders were important for ensuring that new processes were integrated as “business as usual” [33]. Senior leaders were also essential for overall hospital staff involvement and buy-in [33, 37, 50]. The initial decision to begin an implementation effort within the hospital and the subsequent ongoing changes during the implementation process required the engagement of leadership at different levels and from multiple stakeholders across hospital departments [50]. The willingness of middle managers to partake in the implementation process was often contingent upon the support expressed by senior leaders [31]. The absence of senior leader support or tension between middle managers and their direct supervisors meant middle managers were significantly more reluctant to participate. Leaders work to optimize implementation success by expressing enthusiasm for the change; being present, supportive, and attentive to the implementation process; and demonstrating willingness to ask for feedback from staff regarding the change. Leadership that is lacking in authority and unsupportive of change, or that neglects to hold staff accountable for the change, presents barriers to implementation [38, 51]. Staff feel unmotivated to change when leaders were too controlling or unresponsive to requests for more training by staff who were required to implement the practice change [40].

### Resources

This feature is divided into four sub-features that are interrelated and appear to work synergistically to hinder or promote the implementation process.

#### Financial resources

Financial resources were highly important to the implementation process in 17 of 36 studies (47%). Lack of sufficient dedicated funding among acute pain specialized teams meant they struggled to provide adequate service across different departments and sites, leaving no funding reserves for promoting and integrating new innovations. Time that could have been allocated to activities like training and educating staff on the EBP was instead used for seeking funds for other initiatives [51]. Urquhart et al. [52] reported that limited financial resources, including financially dependent resources (e.g., acquiring personnel), were a key constraining feature in implementing a new synoptic reporting tool in different surgery departments. Securing adequate funding to train and educate staff on the new initiative [37–39, 53], allocating human resources to make the change [29, 30, 52], providing monitoring and feedback to ensure fidelity at the change sites [45], and ensuring a smooth transition for the implementation (i.e., new equipment or services to accommodate for the change) [54, 55] were crucial to optimize implementation effectiveness.

#### Staffing and workload

Thirteen of 36 studies (36%) reported on the effects of staffing and workload on the implementation process. Staff experiencing heavy workloads or insufficient staffing on normal routine activities were less likely to carry out change [34, 39, 42, 48, 56, 57]. Assigning dedicated staff to perform the change was associated with successful implementation. Dedicating staff time to implementation activities facilitated effective implementation for pediatric pain management [49] and for obstetrics hemorrhage cases in hospitals [50].

Chuang et al. [31] described how insufficient staffing can be a major implementation challenge for middle managers. Those who could manage staff with little effect on normal working environments were significantly more likely to support the innovation. Middle managers play a key role in facilitating implementation, and their decisions about which staff should undergo training were a key determinant for implementation success [52]. High staff turnover is problematic for implementation, creating a never-ending cycle of training seminars and educational sessions that consume a significant amount of time and resources [38, 53].

#### Time

Thirteen of 36 studies (36%) identified time constraints as a barrier to implementing EBPs. Time constraint was conceptualized at three levels. At the staff level, insufficient time due to other more urgent, competing demands often hindered the full implementation of EBPs [31, 58, 59]. At the innovation level, staff who perceived the EBP as more time-consuming than usual practice were more reluctant to adopt the EBP [57, 59]. Insufficient time for staff training, planning, and staff rescheduling (to implement the EBP) were barriers at the logistics level [36, 38, 49]. Conversely, having adequate time for these activities was positively associated with research use in practice [43, 54, 60].

#### Education and training

Education and training contributed to the effectiveness of EBP implementation in 12 of 36 (33%) studies. Lack of training and development for the EBP among staff and local champions were key barriers to implementation success [38, 39, 51, 53]. Unclear or insufficient educational materials and reminders, inconsistent use of educational materials, and not having enough staff to participate in educational outreach influenced the implementation success of evidence-based pain research in hospital units to varying extents [49]. Staff were more likely to participate in educational sessions and training initiatives if these were offered several times and if leadership mandated the training [53]. Whitley et al. [40] found that high-quality training of a new mental health illness management program by competent and respected trainers was a key factor in high-fidelity sites. Training also promoted interdisciplinary collaboration, since the continuous training sessions provided opportunities for communication and teamwork [52].

### Evaluation, monitoring, and feedback

This feature was important for successful implementation in 14 of 36 studies (39%). Appropriate feedback mechanisms benefited EBP implementation by preserving engagement among staff who implemented the change. Active and engaged leaders who sought feedback about the change and who provided feedback to staff were associated with higher rates of implementation success [33, 34, 53]. Soliciting early feedback from middle managers can help assuage their concerns about the change initiative, and ongoing staff communication and monitoring increased the likelihood of EBP sustainability over time [31]. Three studies reported that evaluation and feedback were important predictors of research use among nurses [32, 54, 60]. Evaluation moderated the effect of nurses’ use of research for pain management [43]. Audit and feedback were effective for improving nursing practice in pain management and assessment for children [49].

### Champions

Presence of champions was important for implementation success in 11 of 36 studies (31%). Champion was the strongest and most consistent feature related to the use of a new systematic framework for prevention delivery services (including the use of implementation guidelines) [45]. Having a champion to advocate for the “new way of doing things” led to more complete and refined use of these guidelines. Supporting champions can be difficult in contexts that lack engaged leadership or dedicated resources to encourage and monitor adherence. Identified champions who rise to the challenge may succumb to feelings of frustrations when the organization does not support change. Key attributes of successful champions included the following: (a) being an expert on the EBP, (b) being available for troubleshooting and for training other staff “on the floor,” and (c) providing a sense of familiarity among colleagues and belief in the champion’s expertise. In one study, the management team chose staff members who were initially unsupportive of the implementation project and motivated them to take an active role in the project, which prevented them from thwarting the implementation progress [53].

### Interrelationships between organizational contextual features

**Fig. 2:**
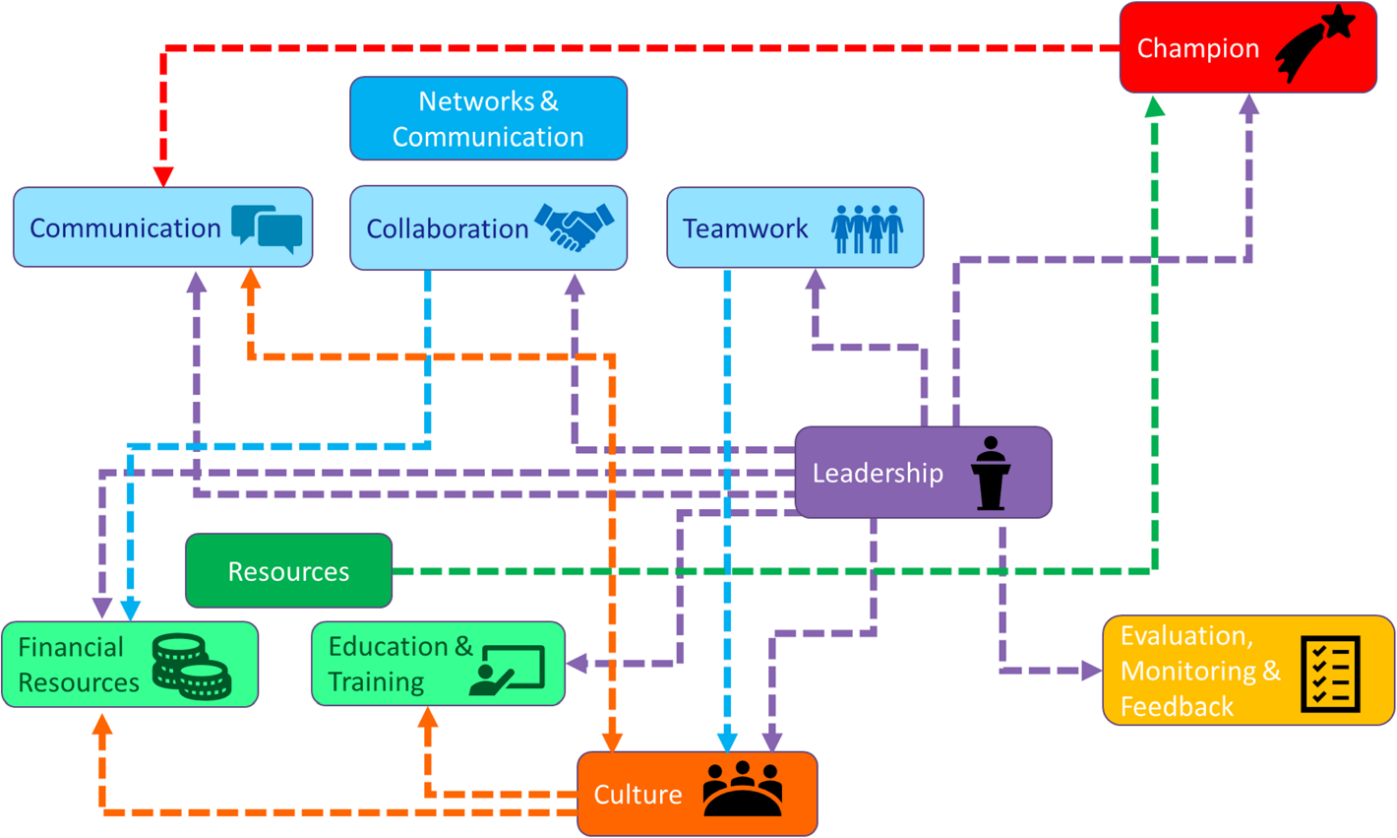
Illustration of the relationships between organizational contextual features and sub-features based on analysis of the results of selected studies. Arrows depict the potential direction of the relationship (e.g., leadership influences evaluation and feedback). The color of each dotted line corresponds to the feature that may exert influence on the other connecting feature. Please refer to the main text for a description of these potential interrelationships between the features and sub-features

Potential interrelationships between features were identified in 12 of 36 studies. Figure 2 illustrates the direction of influence between each feature. Leadership influenced all other features of this review: (a) the use and selection of champions [33, 37, 40, 50], (b) the allocation of resources (funds and additional staffing) to accommodate for the implementation [36–38, 52, 53], (c) the facilitation or hindrance to the monitoring and feedback mechanisms during the implementation process [52, 53], and (d) organizational culture [34, 39, 50, 52].

Networks and communication worked synergistically with other features to promote or hinder the implementation process. For instance, an organizational culture of innovation can be cultivated by ongoing and explicit communication of new innovations [44]. Leaders can help champions communicate observable impacts of the new initiative to other staff, as observed in a study investigating the adoption of clinical practice guidelines in long-term care homes [37]. Similarly, communicating with middle managers and senior leadership to gain buy-in for an EBP was a significant contributor to implementation success [31, 39].

Organizational culture interacted with other organizational contextual features. Bergström et al. [44] found that the organizational culture had set the stage for supportive, inter-professional teamwork and was more important than training staff about implementing the EBP. Sommerbakk et al. [53] demonstrated that a culture characterized by trust and open communication was a facilitator for EBP uptake. Chuang et al. [31] reported that a culture of learning builds teamwork and contributes positively to the performance of the hospital unit that is implementing change. On the contrary, lack of support from colleagues was a barrier to constructing a change culture [53]. Strong leadership, coupled with a culture of learning or openness to innovation, was important to successful implementation [31, 32, 34, 35, 37, 40, 41, 43, 52].

Leadership, culture, resources, and networks and communication contributed to implementation success in at least 50% of the 36 selected studies; among these, 12 studies (33%) identified at least one feature or a sub-feature that influenced or worked synergistically to either act as an enabler or a constraint to the implementation process.

## Discussion

This integrative review identified six organizational contextual features that are important to EBP implementation across healthcare settings. Implementation process is influenced by the organizational culture, leadership, communication and networks, resources, champions, and evaluation, monitoring, and feedback activities within healthcare organizations. Organizational contextual features did not influence implementation efforts independently from other features. Rather, features were interrelated and often influenced each other in complex, dynamic ways to effect change. This finding is congruent with the CFIR, which asserts that the inner setting constructs (discrete theoretical concepts) are interrelated and influence implementation [15]. Given that the six organizational contextual features corresponded to the CFIR inner setting (constructs: culture, networks and communication, resources, leadership engagement) and process (constructs: reflecting and evaluating, champions) domains, the CFIR may serve as an appropriate framework for assessing or improving organizational context in a wide range of healthcare settings. Notably, the CFIR identified 39 constructs, which go well beyond the six features that were identified from this review. Identifying the most commonly reported features that influence the implementation provides preliminary evidence that these may be the most important for optimizing implementation effectiveness.

Kirk et al. [61] and May et al.’s [62] systematic review of studies that used the CFIR as a guiding framework found variation in the use of CFIR constructs, but these studies rarely justified their choice. Justifying which CFIR constructs to investigate can help ensure the consistency of implementation studies and allows researchers to compare these studies over time and across different settings [15, 61, 62]. Findings of this review can provide preliminary guidance for selecting which contextual features to modify during implementation planning. Nonetheless, readers should keep in mind how these contextual features were identified in the included studies. Over half (56%) of the included studies were guided by frameworks and measures or applied existing theoretical perspectives. Researchers of these included studies may be sensitized to specific contextual features or constructs, which may have precluded examination of other features beyond those illustrated in the guiding theory, framework, or model. Many included studies did not define organizational context, and those that were guided by frameworks, theories, or models conceptualized organizational context differently.

Without a single operational definition, studies claiming to investigate organizational context may be examining different constructs. Researchers suggested that incomplete definitions of context, combined with inconsistencies in definitions, have led to conceptual overlap and confusion in the specification of context [63, 64]. Measuring and assessing a core set of contextual features across healthcare settings can allow for a synthesis of findings across studies to detect trends that consistently influence implementation outcomes. By operationally defining organizational context, implementation researchers can advance the existing limited evidence base on understanding how contextual features can affect implementation and under which conditions. Findings of this review can provide some indication on how implementation health researchers are operationally defining organizational context.

The six contextual features combine to promote or hinder implementation depending on their presence or absence. Organizations that comprise low-fidelity implementation sites usually involve the absence or malfunction of one or more of these features. Capitalizing on these features most likely supports implementation activities. The finding that organizational contextual features synergistically influenced implementation efforts supports that context is not just a physical setting or a backdrop for implementation; organizational contextual features interact, impact, modify, promote, or hinder the EBP and its implementation efforts. Furthermore, the EBP, target users, implementation process, and inner and outer contexts are intertwined, constantly interacting with and influencing each other [15]. The interrelationships between organizational contextual features support Aarons et al.’s [65] postulation that context should not be viewed as a fixed, organizational structure or institutional entity but as an unstable, unfolding process.

Organizational culture was most commonly reported to affect EBP implementation. However, extant literature suggests very limited, if any, interventions to improve organizational culture in healthcare settings [66]. Culture exerts influence on available resources such as funding and educational support, and can be modified by the type of leadership (i.e., transformational versus authoritarian), level of communication (i.e., low versus high), and quality of teamwork within the organization. One study examining mental health clinician attitudes on EBPs found that more engaged organizational cultures and implementation climates, characterized by higher levels of educational support, coupled with more interactive implementation leadership were associated with more positive attitudes toward EBPs [67]. The researchers examined implementation-specific organizational constructs (e.g., implementation climate, implementation leadership) that are more proximal to implementation. The relationship between general organizational context (as reviewed in this paper) and implementation-specific organizational context has yet to be fully established. Future implementation strategies should address features that are associated with both general and implementation-specific organizational contexts to explore their potential roles as mediators and moderators of implementation effectiveness [68].

Leadership influences all other features, suggestings that it may be a priority feature in implementation efforts. Empirical evidence supports the critical importance of leaders in the implementation process [69, 70] and considers leadership as essential for creating an organizational context conducive to change [71, 72]. There is a need to better understand how leadership interacts with other key features associated with implementation success so that resources can be meaningfully directed to shape the contextual features that have high impact on implementation outcomes.

### Limitations

This review was limited to published journal articles in English; the results may have limited transferability to non-English-speaking nations that have very different healthcare systems. This review was also limited to studies that investigated organizational contextual features during the implementation, adoption, and uptake phases of EBPs; these studies provided little understanding of how organizational contextual features impact the sustainability of EBPs. The search strategy of this review used the term “context” in the organization to identify empirical studies that investigated organizational context. However, it is likely that other researchers who examined the same organizational contextual features identified in this review may not use the term “context” in their report. As such, these studies could not be retrieved. For example, Williams et al. [73] reported an increase in EBP uptake through improved organizational culture among mental health clinicians in 14 children’s mental health agencies. This study was not captured in this review but proves to be highly relevant to inform implementation researchers about the value of organizational culture change on implementation effectiveness. Guerrero et al. [74] observed that the leader’s openness to and expectations about implementing EBPs were strongly associated with the implementation of a contingency management strategy in substance abuse treatment programs. Therefore, readers should approach the review findings with caution, bearing in mind the limitations of the search strategy in this review.

Several limitations at the level of individual studies warrant discussion. This review identified potential interrelationships between the organizational contextual features but did not explore the nature of these relationships, with one exception [46]. Study findings were reported very briefly in the “Results” sections, which precluded reviewers from drawing further conclusions about these interrelationships. The extent to which these features may be more effective for implementation if considered in concert or individually remains an empirical question that needs further exploration. The organization contextual features identified as consistently influential to implementation efforts were contingent upon the study authors’ decisions as to which features belonged at the organizational level. It is possible that other less frequently explored contextual features can also influence implementation outcomes.

Although the term “implementation success” frequently appeared in the “Results” and “Discussion” sections of the included studies, this term was not defined. Implementation success can be measured or conceptualized differently in different healthcare settings. Implementation studies should describe how “implementation success” is conceptualized or operationalized in the implementation project, or report on any pre-determined targets that represent implementation effectiveness. Most of the included studies used qualitative approaches to identify, describe, or explain the organizational contextual features that emerged from this review; however, it was unclear whether the conceptual or operational definitions for each of these features (e.g., culture, leadership) were consistent across the included studies. Defining each feature being investigated will enhance the clarity and consistency of the feature and facilitate external validity.

Even though 27 out of 36 included studies were rated as moderately high to high quality according to the MMAT, the included studies did not follow any standard methods of reporting, which is consistent with existing literature that articulated the low reporting standards of implementation studies [74, 75]. Implementation researchers should consider using the Standards for Reporting Implementation Studies (StaRI) [76] to ensure transparent and accurate reporting of implementation studies. StaRI requires researchers to provide an extensive description of context, which will help readers assess the external validity of the reported study, and decide how the implementation context in the study compares to their own setting. A rich description of the study’s implementation context is crucial to readers who are considering whether the implementation strategy can be directly adopted or will need modifications [77].

## Conclusions

This integrative review provides an overview of how implementation researchers operationalized organizational context in healthcare settings, and describes the potential interrelationships among the six most commonly reported organizational contextual features that influence EBP implementation. Shared commonality in how we define, assess, and measure organizational context can add to the generalizability of future studies. A core set of organizational contextual features influencing the implementation of EBPs exist across a wide range of healthcare settings. These organizational contextual features were consistent with the constructs illustrated in CFIR [15], supporting its use as a guiding framework for exploring the relationship between organizational contextual features and implementation. Future research needs to confirm this finding and examine the interrelationships between different contextual features which, by working together, can act as enablers in one implementation setting but barriers in others. Accounting for interconnections among organization contextual features at each KT phase may enable implementation researchers to more fully describe the determinants of successful implementation in clinical practice. Developing a conducive organizational context, specifically with strong leadership capacity, can be an essential precursor to facilitate the implementation of EBPs in a wide range of healthcare settings.

## Additional file

Additional file 1: Literature search strategy (MEDLINE). (DOCX 13 kb)

## Abbreviations

CFIR: Consolidated Framework for Implementation Research
CINAHL: Cumulative Index to Nursing and Allied Health Literature
EBP: Evidence-based practice
EMBASE: Excerpta Medica Database
HCP: Healthcare professional
ICU: Intensive care unit
KT: Knowledge translation
MEDLINE: Medical Literature Analysis and Retrieval System Online
MMAT: Mixed Methods Appraisal Tool
PARiHS: Promoting Action Research in Health Services
PRISMA: Preferred Reporting Items for Systematic Reviews and Meta-Analyses
PsycINFO: American Psychological Association’s (APA) resource for abstracts of scholarly journal articles, book chapters, books, and dissertations
StaRI: Standards for Reporting Implementation Studies
