# Supplementary material for: Organizational contextual features that influence the implementation of evidence-based practices across healthcare settings: a systematic integrative review
Source: Syst Rev. 2018 May 5;7:72. doi: 10.1186/s13643-018-0734-5 (PMC5936626; doi:10.1186/s13643-018-0734-5)
Supplement: Supplementary file 1 — Literature search strategy (MEDLINE). (DOCX 13 kb) [file 13643_2018_734_MOESM1_ESM.docx]

Additional File 1

MEDLINE search strategy

| 1. Knowledge translation.tw,hw,sh,fs,kw,ot. or exp Translational Medical Research/ |
| --- |
| 2. (knowledge adj3 (transfer* or translat* or disseminat* or exchange or broker* or use* or adopt* or uptak*)).tw,hw,sh,fs,kw,ot. |
| 3. (implement* adj3 (science or research)).tw,hw,sh,fs,kw,ot. |
| 4. (research adj3 (adopt* or uptak* or use* or utili?ation*)).tw,hw,sh,fs,kw,ot. |
| 5. exp "Diffusion of Innovation"/ |
| 6. Information dissemination/ |
| 7. (research adj3 (adopt* or disseminat* or transfer* or translat* or mobili?ation or exchange or utili?ation or diffusion or uptak* or use*)).tw,hw,sh,fs,kw,ot. |
| 8. 1 or 2 or 3 or 4 or 5 or 6 or 7 |
| 9. context*.tw,hw,sh,fs,kw,ot. |
| 10. 8 and 9 |
| 11. (Meta-Analysis as Topic/ or meta analy$.tw. or metaanaly$.tw. or Meta-Analysis/ or (systematic adj (review$1 or overview$1)).tw. or exp Review Literature as Topic/ or cochrane.ab. or embase.ab. or (psychlit or psyclit).ab. or (psychinfo or psycinfo).ab. or (cinahl or cinhal).ab. or science citation index.ab. or bids.ab. or cancerlit.ab. or reference list$.ab. or bibliograph$.ab. or hand-search$.ab. or relevant journals.ab. or manual search$.ab. or ((selection criteria or data extraction).ab. and review/)) not (comment/ or letter/ or editorial/) |
| 12. 10 not 11 |
| 13. limit 12 to journal article |
| 14. limit 13 to english language |
| 15. limit 14 to human |
| 16. limit 15 to yr="2005 -Current" |
| 17. limit 16 to humans |
| 18. remove duplicates from 17 |
| 19. organi?ation*.tw,kw,ot. |
| 20. 18 and 19 |
